# Protective Effect of Patchouli Alcohol Against High-Fat Diet Induced Hepatic Steatosis by Alleviating Endoplasmic Reticulum Stress and Regulating VLDL Metabolism in Rats

**DOI:** 10.3389/fphar.2019.01134

**Published:** 2019-10-01

**Authors:** Xue Wu, Nan Xu, Minyao Li, Qionghui Huang, Jiazhen Wu, Yuxuan Gan, Liping Chen, Huijuan Luo, Yucui Li, Xiaoqi Huang, Ziren Su, Yuhong Liu

**Affiliations:** ^1^Guangdong Provincial Key Laboratory of New Drug Development and Research of Chinese Medicine, Mathematical Engineering Academy of Chinese Medicine, Guangzhou University of Chinese Medicine, Guangzhou, China; ^2^The First Affiliated Hospital of Chinese Medicine, Guangzhou University of Chinese Medicine, Guangzhou, China; ^3^Sun Yat-Sen Memorial Hospital, Sun Yat-Sen University, Guangzhou, China

**Keywords:** patchouli alcohol, nonalcoholic fatty liver disease, hepatic steatosis, very low-density lipoprotein, endoplasmic reticulum stress

## Abstract

Nonalcoholic fatty liver disease (NAFLD) is currently the most common chronic hepatic disorder worldwide. The earliest stage of NAFLD is simple steatosis, which is characterized by the accumulation of triglycerides in hepatocytes. Inhibition of steatosis is a potential treatment for NAFLD. Patchouli alcohol (PA) is an active component of *Pogostemon cablin* (Blanco) Benth. (Labiatae), which is a medicinal food in Asia countries and proved to possess hepatoprotective effect. This research aimed to investigate the effectiveness of PA against high fat diet (HFD)-induced hepatic steatosis in rats. In this study, male Sprague Dawley rats were fed a HFD for 4 weeks to induce NAFLD. Oral administration with PA significantly reduced pathological severity of steatosis in HFD-fed rats. It was associated with suppressing endoplasmic reticulum (ER) stress and regulating very low-density lipoprotein (VLDL) metabolism. Our data showed that PA treatment effectively attenuated ER stress by inhibiting the activation of protein kinase-like ER kinase (PERK), inositol-requiring transmembrane kinase/endoribonuclease 1 (IRE1), and activating transcription factor 6 (ATF6). Moreover, PA decreased hepatic VLDL uptake by suppressing very low-density lipoprotein receptor (VLDLR) expression. It also restored VLDL synthesis and export by increasing apolipoprotein B100 (apoB 100) secretion and microsomal triglyceride-transfer protein (MTP) activity. Taken together, PA exerted a protective effect on the treatment of NAFLD in HFD-fed rats and may be potential therapeutic agent acting on hepatic steatosis.

**Graphical Abstract:**
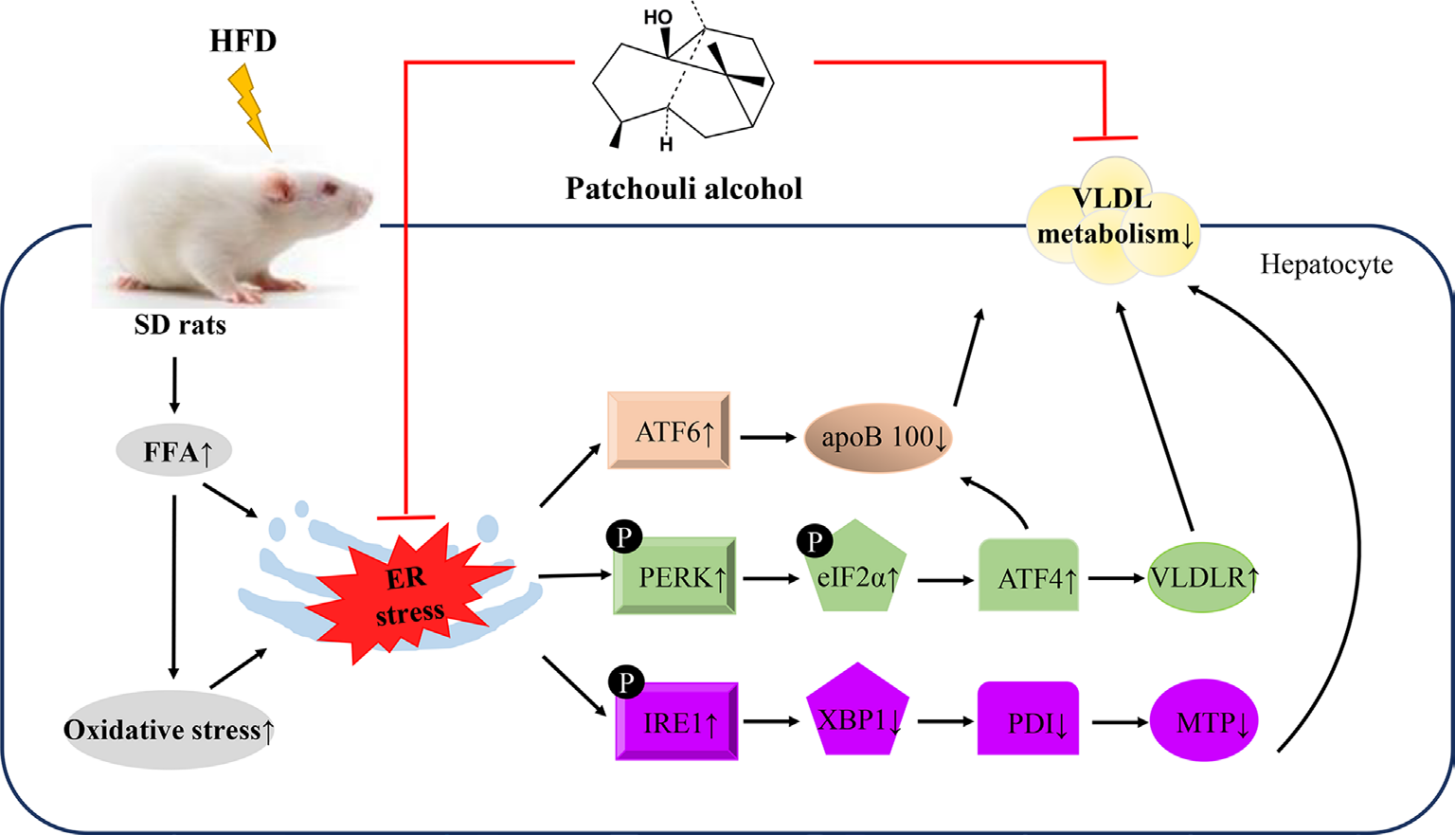
This study demonstrated that supplementation of PA attenuated HFD-induced hepatic steatosis by suppressing ER stress and regulating VLDL metabolism in rats.

## Introduction

Nonalcoholic fatty liver disease (NAFLD), a result of the pandemic of obesity and diabetes, has become a leading cause of liver disease and emerged a major challenge in modern society. It affects 6–45% of the general population worldwide with highly prevalence and incidence (Fazel et al., 2016). NAFLD is one of the most common chronic liver diseases, ranging from simple steatosis to nonalcoholic steatohepatitis (NASH), and eventually to hepatocellular carcinoma (HCC) (Younossi et al., 2019). Hepatic steatosis occurs in the early stages of NAFLD, which is defined as abnormal lipid deposition in the liver. When the rate of hepatic lipid uptake exceeds the rate of lipid disposal, lipid accumulates in the liver and results in steatosis (Ipsen et al., 2018). Very low-density lipoprotein (VLDL) metabolism is considered as a beneficial factor in overcoming the excess formation of triglyceride (TG) and regulating intrahepatic and plasma lipid homeostasis. A dysregulation in VLDL uptake, export, or synthesis is one of the major causes of hepatic steatosis (As, 2018). Current studies have demonstrated that endoplasmic reticulum (ER) stress is implicated in the inhibition of VLDL metabolism. ER stress interferes with VLDL metabolism in several manners, including enhancing VLDL delivery to hepatocytes and inhibiting VLDL synthesis and export from these cells. Free fatty acids (FFAs) from diet are thought to be crucial for the onset of hepatic steatosis. Elevated level of FFAs results in disruption of ER homeostasis and VLDL metabolism, bringing about TG accumulation in the liver and finally leading to hepatic steatosis (Wei et al., 2007; Ghemrawi et al., 2018).

**Figure 1:**
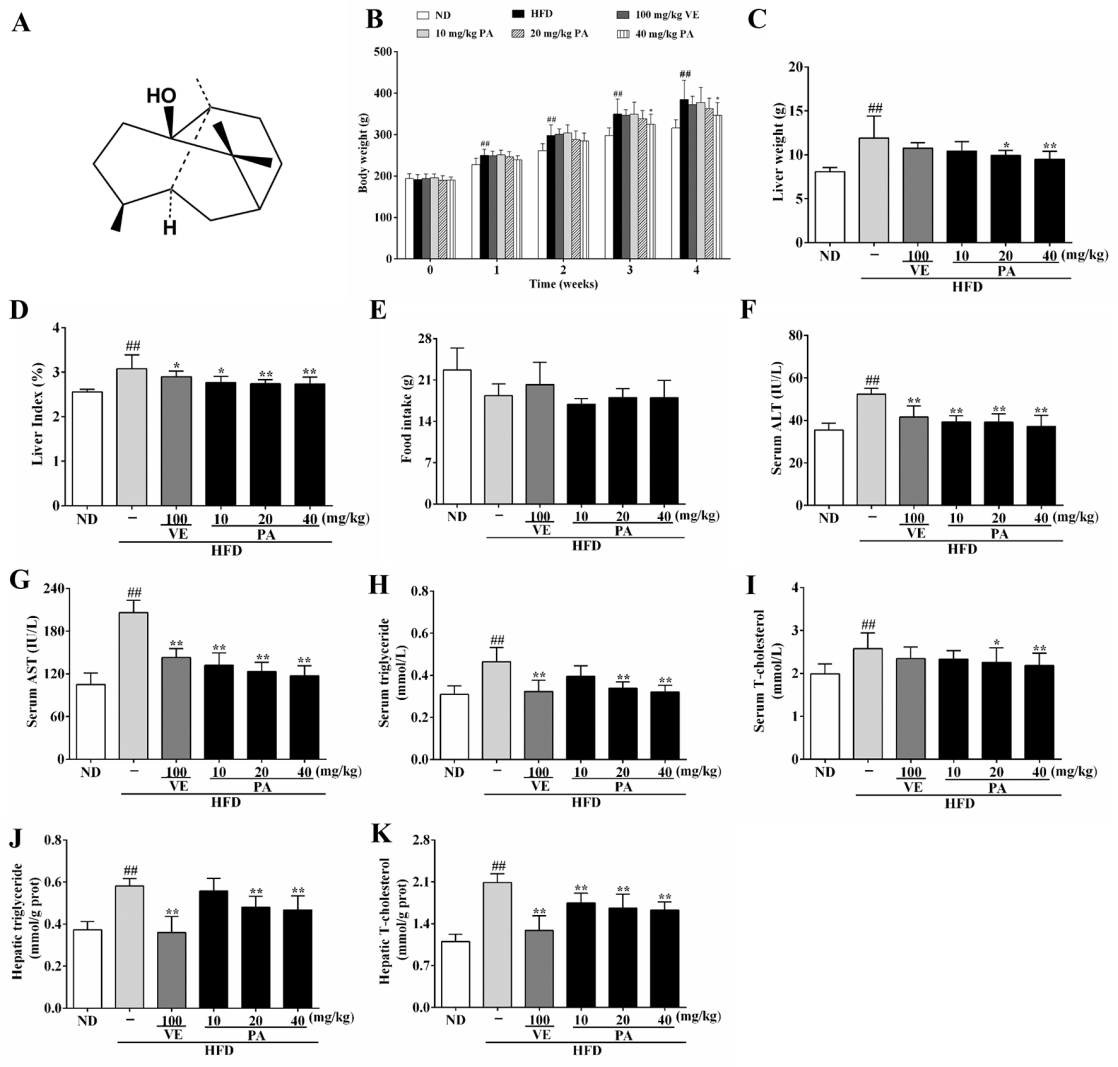
Chemical structure of patchouli alcohol (PA) and PA treatment attenuated HFD-induced lipid accumulation in rats. **(A)** Chemical structure of PA; **(B)** Body weight; **(C)** Liver weight; **(D)** Liver index; **(E)** Food intake; **(F)** Serum levels of ALT, **(G)** AST, **(H)** TG and **(I)** TC; **(J)** Hepatic levels of TG and **(K)** TC. Values were presented as mean ± SD (*n* = 8 per group). ^##^ *p* < 0.01 vs. ND group; *p < 0.05,**p < 0.01 vs. HFD group.

To date, there are no effective medical intervention strategies for NAFLD. Natural compounds are deemed as viable treatment regimens to inhibit the progress of NAFLD because of the beneficial effects they have shown (Li et al., 2018; Zhu et al., 2018). *Pogostemon cablin* (Blanco) Benth. (Labiatae) is a widely used traditional healthy food and medicinal herb in Asian countries such as China, Malaysia, and India. Its fresh leaves and dried powder are used in the form of food flavour supplements, tea, beverages, candy, baked products, and common botanical ingredients in functional foods and dietary supplements (Hou and Jiang, 2013; Preedy, 2016). It has been reported to display excellent anti-inflammatory, anti-oxidative, and multiple-organ protective activities (Zhang et al., 2016; Chen et al., 2017). Our previous study also confirmed the protective effect of patchouli oil (the extractive from the dry leaves of *Pogostemon cablin*) against lipid accumulation in a rat model of alcoholic liver injury (ALI) (Huang et al., 2018). Patchouli alcohol (PA, **Figure 1A**), as the phytochemical marker determining the quality of *Pogostemon cablin* and patchouli oil, has been demonstrated to possess various medicinal activities (Yu et al., 2015; Liu et al., 2017). However, the mechanism of PA action in NAFLD by reducing hepatic steatosis still remains uncertain. Therefore, this study aimed to evaluate if PA supplementation to a HFD would reduce hepatic steatosis by alleviating ER stress and regulating VLDL metabolism in rats.

## Materials and Methods

### Drugs and Chemicals

PA was isolated from patchouli oil according to published article at purity of 99.0% (Su et al., 2014). Vitamin E (VE; purity ≥ 98%) was purchased from Dalian Meilun Biological Technology Co. Ltd (Dalian, Liaoning, China). Normal diet (ND; ≥ 4% energy as fat) was purchased from the Medical Experiment Animal Center of Guangzhou University of Chinese Medicine, and high fat diet (HFD, D12492, 60% energy as fat) was purchased from Guangdong Medical Lab Animal Center (Guangzhou, Guangdong, China). The kits for biochemical analysis of aspartate transaminase (AST), alanine aminotransferase (ALT), triglyceride (TG), total cholesterol (TC), superoxide dismutase (SOD), glutathione (GSH) catalase (CAT), and malondialdehyde (MDA) were obtained from Nanjing Jiancheng Bioengineering Institute (Nanjing, Jiangsu, China). ELISA kits for free fatty acid (FFA), reactive oxygen species (ROS), apolipoprotein B100 (apoB 100), and VLDL measurement were purchased from Shanghai Enzyme-linked Biotechnology Co. Ltd (Shanghai, China). Primers for determination of mRNA expressions of glucose-regulated protein 78 kDa (GRP78), protein kinase-like ER kinase (PERK), inositol-requiring transmembrane kinase/endoribonuclease 1 (IRE1), eukaryotic translation initiation factor 2α (eIF2α), activating transcription factor 4 (ATF4), very low-density lipoprotein receptor (VLDLR), X box binding protein 1 (XBP1), protein disulfide isomerase (PDI), microsomal triglyceride-transfer protein (MTP), activating transcription factor 6 (ATF6), apoB 100, and glyceraldehyde-3-phosphate dehydrogenase (GAPDH) were provided by Sangon Biotech Co., Ltd (Shanghai, China). Primary antibodies against GRP78 (AF5366), PERK (AF5304), p-PERK (DF7576), eIF2α (AF6087), p-eIF2α (AF3087), IRE1 (DF7709), p-IRE1 (DF8322), ATF4 (DF6008), XBP1 (AF5110), MTP (DF6591), ATF6 (DF6009), β-actin (AF7018), and goat anti-rabbit IgG (H+L) HRP (S0001) secondary antibody were obtained from Affinity Biosciences Inc. (USA). Primary antibody against VLDLR (19493-1-AP) was purchased from Proteintech Group Inc. (USA). Primary antibody against PDI (CY6636) was purchased from Abways Technology Inc. Primary antibody against apoB 100 (A1330) was purchased from ABclonal Technology Inc. (USA). All reagents were of analytical grade.

### Animals and Treatments

Animal experiment procedures were approved by the Ethics Committee for the Welfare of Experimental Animals of Guangzhou University of Chinese Medicine (No. 20181015002). Male Sprague Dawley rats were purchased from the Medical Experiment Animal Center of Guangzhou University of Chinese Medicine (SYXK (YUE) 2018-0085). After 7 days acclimation, rats were randomly divided into six experimental groups (*n* = 8): ND, HFD, HFD supplemented with VE (100 mg/kg), and HFD supplemented with different doses of PA (10, 20, and 40 mg/kg), respectively. VE was used as positive control. PA and VE were dissolved in 0.5% tween 80 solution. Except the ND group (fed with normal diet), all rats were fed with HFD and water *ad libitum* for a period of 4 weeks to induce NAFLD. Meanwhile, rats received intragastric administration once daily. The body weights of rats were recorded daily, and food intake were recorded every week throughout the study. At the last day, all rats were weighted and anesthetized with sodium pentobarbital after fasting overnight. Blood samples from each rat were collected for biochemical analysis and their livers were rapidly dissected for histological evaluation and further analysis.

### Serum Biochemistry

Blood samples were centrifuged at 3000 rpm at 4°C for 10 min, and the supernatants were collected for serum biochemistry examination. The serum levels of ALT, AST, TG, and TC were measured with commercial assay kits according to manufacturer’s instructions by microplate reader.

### Histopathological Analysis

After fixed with 4% paraformaldehyde, liver segments were dehydrated, cleaned, and embedded in paraffin. Then, the liver slices of 5 μm thickness were stained with hematoxylin and eosin (H&E) and 5-μm-thick frozen sections were stained with Oil red O. Analysis were performed under a light microscope according to the method previously reported (Ding et al., 2017; Huang et al., 2017).

### Hepatic Biochemical Analysis

The liver tissues were homogenized in ice-physiological saline or absolute ethanol, and centrifuged at 4000 rpm and 4°C for 10 min to obtain supernatant for further analysis. Liver SOD, GSH, CAT, MDA, TG, and TC levels were determined using commercial assay kits according to corresponding product specifications and analyzed by microplate reader.

### Enzyme Linked Immunosorbent Assay (ELISA)

Liver tissues were homogenized in phosphate buffer saline (pH 7.2–7.4) and then centrifuged at 4000 rpm at 4°C for 10 min to obtain supernatants for hepatic FFA, ROS, and VLDL examination. Blood samples were centrifuged at 3000 rpm at 4°C for 10 min, and the supernatants were collected for serum VLDL and apoB 100 examination. Hepatic levels of FFA, ROS, and VLDL as well as serum levels of VLDL and apoB 100 were measured by ELISA kits using a microplate reader.

### Western Blot Analysis

Hepatic proteins were extracted using a commercial protein extraction kit (Servicebio, Wuhan, Hubei, China). After denaturation, proteins were separated by electrophoresis on SDS-PAGE gels and transferred to PVDF membranes. Membranes were blocked for 1 h with 5% (w/v) skim milk in Tris-buffered saline-tween 20 (TBST) and incubated with 1: 1000 dilution of primary antibodies and 1:3000 dilution of HRP-conjugated secondary antibody. Protein bands were visualized with ECL reagents (AmershamBiosciences, Buckinghamshire, UK), and densitometry analysis was performed using Quantity One 4.6.2 software. All blots were quantified and normalized against β-actin to adjust for the amount of proteins loaded.

### Reverse Transcription-Quantitative Polymerase Chain Reaction (RT-qPCR)

Total RNA was extracted from liver tissue using TRI-zol^®^ reagent and further synthesized as cDNA. The cDNA was used as the template for real-time PCR. The mRNA expressions of GRP78, PERK, eIF2α, IRE1, ATF4, VLDLR, XBP1, PDI, MTP, ATF6, and apoB 100 (the primers of PCR are listed in **Table 1**) were measured by HiScript^®^ II Q RT SuperMix (+gDNA wiper) and ChamQ^™^ SYBR^®^ qPCR Master Mix Kit according to manufacturer’s instruction (Vazyme Biotech, China). Each reaction was conducted in triplicate under the following cycling conditions: 10 min at 95°C, followed by 40 cycles at 95°C for 15 s and then 60°C for 1 min. The relative expression levels of target genes were normalized by GAPDH. The relative quantification of gene expression was calculated using the 2^−ΔΔCt^ method.

**Table 1:** Primer sequences.

Gene	Forward primer (5’-3’)	Reverse primer (5’-3’)
GRP78	CGGAGGAGGAGGACAAGAAGGAG	ATACGACGGTGTGATGCGGTTG
PERK	CGCTGCTGCTGCTGTTCCTG	GCAATGCCTCGGCGTCTTCC
IRE1	GACGAGCATCCGAATGTGATCCG	GAGGTGGTCTGATGAAGCAAGGTG
ATF6	GGCTTCCTCCAGTTGTTCTGTCTC	GCTTCTCTTCCTTCAGTGGCTCTG
eIF2α	GCCGATAAGGTTACGATGCTGTGG	GTAGGAAGCGCCTGTCTTGTCAAC
ATF4	GACCGAGATGAGCTTCCTGAACAG	CCGCCTTGTCGCTGGAGAAC
VLDLR	GACGCAGACTGTTCCGACCAATC	GCAGGTTCGAGAAGGACAGTTGAC
apoB 100	TCTGACTGGTGGACTCTGACTGC	TCTTGGAGAGCGTGGAGACTGAC
XBP1	AGGTCTCAGAGGCAGAGTCCAAG	AAGAGGCAACAGCGTCAGAATCC
PDI	CAACGTCCTGGTGCTGAAGAAGAG	TGCTAGTCGGATCTCAGAGCCTTC
MTP	TTCATTCAGCACCTCCGCACTTC	AGTCCAGGATGGCTTCCAGTGAG
GAPDH	ACGGCAAGTTCAACGGCACAG	CGACATACTCAGCACCAGCATCAC

### Statistical Analyses

Data were presented as mean ± SD. Statistical analyses were performed using Statistical Product and Service Solutions (SPSS) software (version 20.0). Group differences were assessed by one-way analysis of variance (ANOVA) followed by an LSD test for multiple comparisons. *P* < 0.05 was considered statistical significant.

## Results

### Effect of PA on Body Weight, Liver Weight, Liver Index, and Lipid Accumulation in HFD-Fed Rats

**Figure 2:**
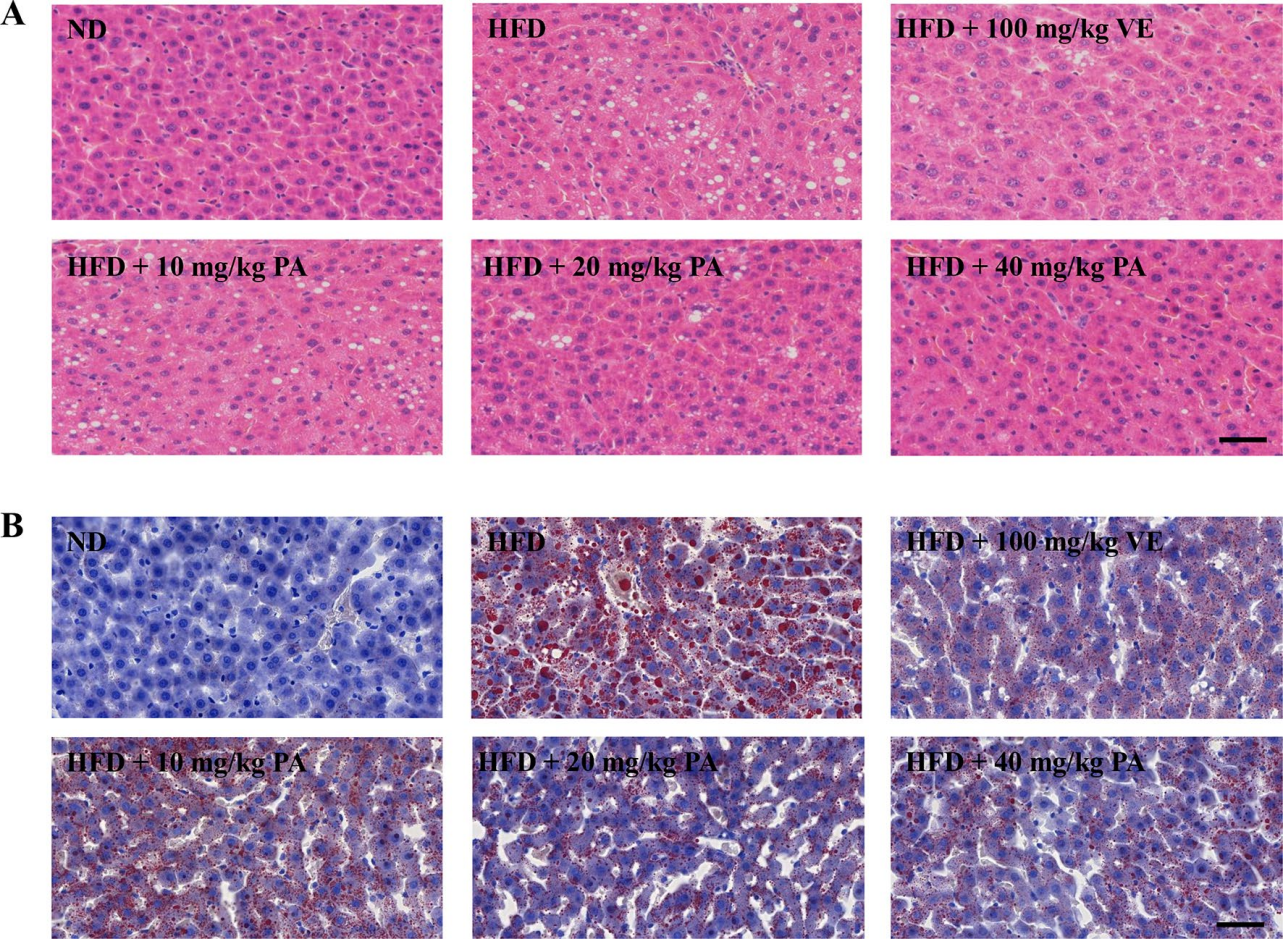
PA treatment attenuated HFD-induced hepatic steatosis in rats. **(A)** Representative photomicrographs of H&E staining (400×) and **(B)** Oil Red O staining (400×) of livers.

Chronic exposure to HFD disturbed lipid homeostasis in a time-dependent manner leading to development of NAFLD. Data in **Figures 1B–D** showed that a profound increase in body weight, liver weight, and liver index was observed in the HFD group. VE treatment did not alter the increase of body weight and liver weight, but significantly decreased liver index in rats. Furthermore, PA treatment dramatically reduced body weight, liver weight, and liver index in HFD-fed rats. Data in **Figure 1E** showed that there was no significant difference in the food intake among all groups. As shown in **Figures 1F–K**, compared to the rats only fed with ND, the serum levels of AST, ALT, TG, and TC along with hepatic levels of TG and TC were significantly increased in the HFD-fed rats. These lipid parameters in VE and PA treated groups were lower than the HFD group. Moreover, rats with 40 mg/kg PA treatment exhibited superior effect in the reduction of serum ASL, ALT, TG, and TC among all treated groups. Histological assessments of liver tissue in **Figure 2** showed presence of NAFLD, characterized by increased vesicular lipid droplets, hepatic vacuoles, and slight inflammatory infiltrate in HFD-fed rats. VE and PA supplementation for 4 weeks markedly decreased vacuoles, lipid droplets area, and inflammation in the liver of HFD-fed rats. These results indicated that PA was effective in reducing HFD-induced body weight gain and preventing hepatic steatosis in rats.

### Effect of PA on FFA and Oxidative Stress in HFD-Fed Rats

**Figure 3:**
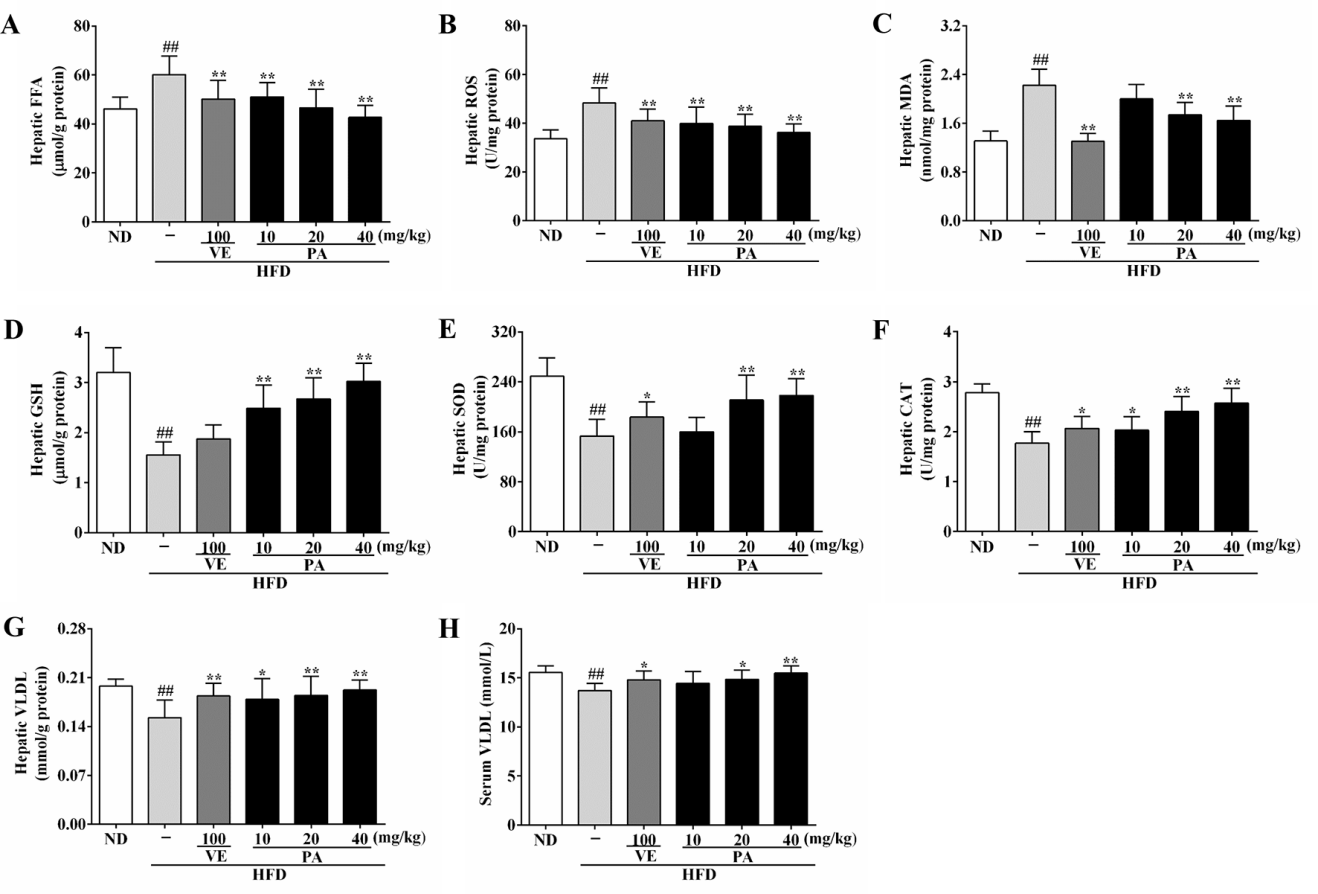
PA treatment reduced HFD-induced elevated FFA level, oxidative stress, and decreased VLDL levels in rats. **(A)** Hepatic levels of FFA, **(B)** ROS, **(C)** MDA, **(D)** GSH, **(E)** SOD, **(F)** CAT, and **(G)**VLDL; **(H)** Serum level of VLDL. Values were presented as mean ± SD (*n* = 8 per group). ^##^ *p* < 0.01 vs. ND group; *p < 0.05,**p < 0.01 vs. HFD group.

ER stress is often associated with FFA and oxidative stress. To explore the role of PA against elevated FFA and oxidative stress, we examined the hepatic levels of FFA and the oxidative stress indicators: ROS and MDA. As shown in **Figures 3A–C**, higher levels of FFA, ROS, and MDA were presented in the HFD-fed rats when compared to the ND-fed rats. However, the PA or VE groups showed similar hepatic levels of FFA, ROS, and MDA to ND group when compared to the HFD group. Data in **Figures 3D–F** showed that the hepatic levels of GSH, SOD, and CAT were lower in the HFD-fed rats than ND-fed rats. Conversely, VE or PA supplementation dramatically increased the activities of SOD and CAT in HFD-fed rats. Moreover, the level of GSH was normalized in VE or PA treated rats, whereas there was no significant difference between HFD-fed rats and VE treated rats. Collectively, these results indicated that PA exerted a protective effect against elevated FFA and oxidative stress in HFD-fed rats.

### Effect of PA on ER Stress and VLDL Secretion in HFD-Fed Rats

**Figure 4:**
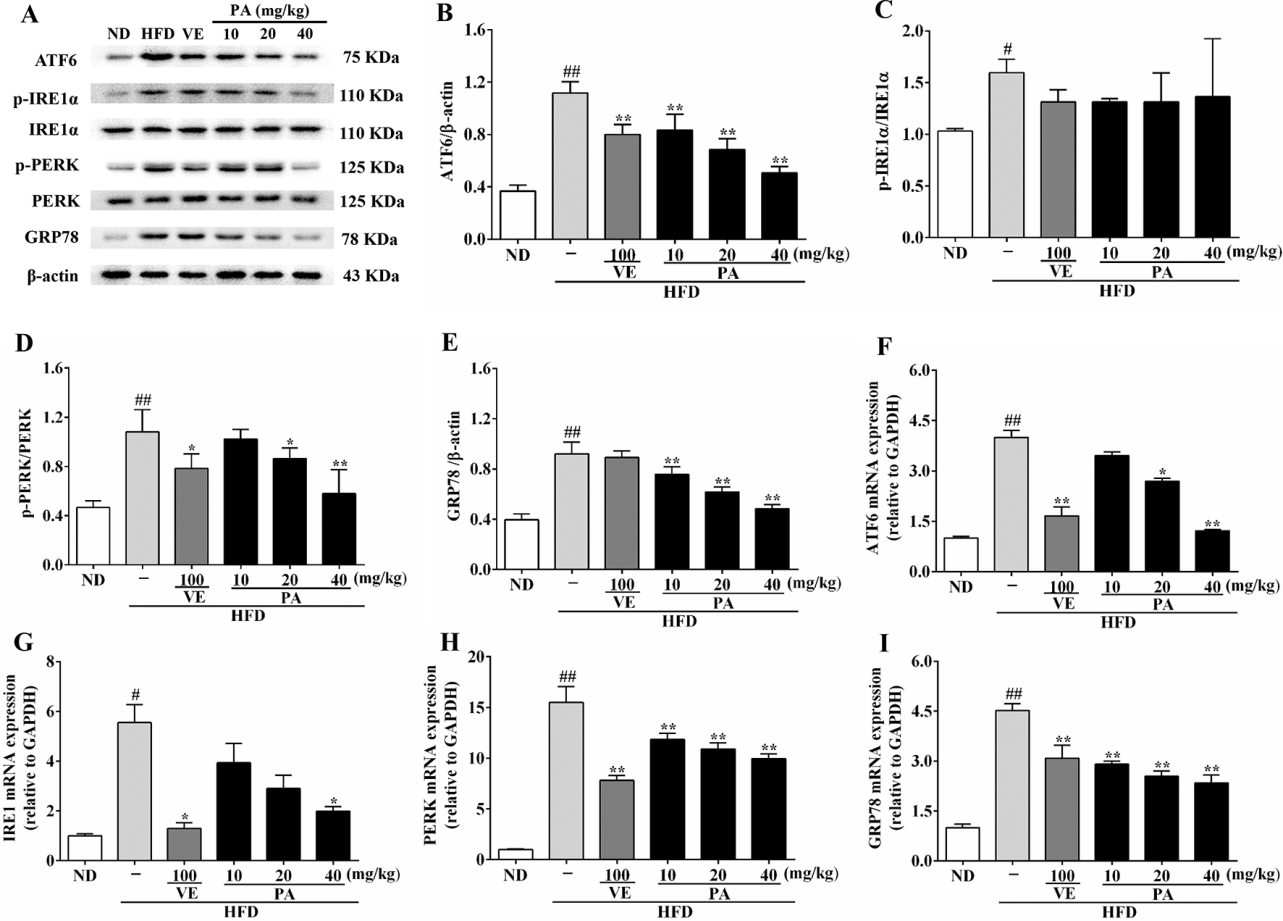
PA treatment attenuated HFD-induced ER stress in rats. **(A)** Representative immunoreactive bands of GRP78, PERK, p-PERK, IRE1α, p-IRE1α, and ATF6; **(B)** Ratios of ATF6/β-actin, **(C)** p-IRE1α/IRE1α, **(D)** p-PERK/PERK, and **(E)** GRP78/β-actin; the relative expression levels of target proteins were normalized by β-actin; **(F)** mRNA expressions of ATF6, **(G)** IRE1, **(H)** PERK, and **(I)** GRP78; the relative expression levels of target genes were normalized by GAPDH. Values were presented as mean ± SD (*n* = 3 per group). *p* < 0.05, ^##^ *p* < 0.01 vs. ND group; *p < 0.05, **p < 0.01 vs. HFD group.

The enhanced FFA and oxidative stress induced by HFD-fed resulted in aggravating ER stress and decreasing VLDL secretion. As shown in **Figures 3G and H**, HFD feeding decreased the hepatic and serum levels of VLDL; however, VE or PA treated rats exhibited higher hepatic and serum levels of VLDL than HFD-fed rats. Data in **Figure 4** indicated that HFD-induced ER stress markers, including GRP78, PERK, IRE1, and ATF6, were inhibited by VE or PA supplementation. Data in **Figures 4A–E** showed that HFD-fed rats significantly increased hepatic protein expressions of GRP78 and ATF6. These ER markers were down regulated by VE or PA treatment, whereas VE-treated rats had no significant change on GRP78 expression. In addition, VE or PA treatment decreased the ratios of p-PERK/PERK and p-IRE1α/IRE1α in HFD-fed rats, whereas there was no significant difference in the ratio of p-IRE1α/IRE1α between HFD and the treated groups. Data in **Figures 4F–I** showed that the hepatic mRNA expressions of GRP78, PERK, IRE1, and ATF6 were dramatically decreased after administration with PA and VE. These results indicated that PA was a regulator for ER homeostasis and VLDL secretion.

### Effect of PA on VLDLR Expression in HFD-Fed Rats

**Figure 5:**
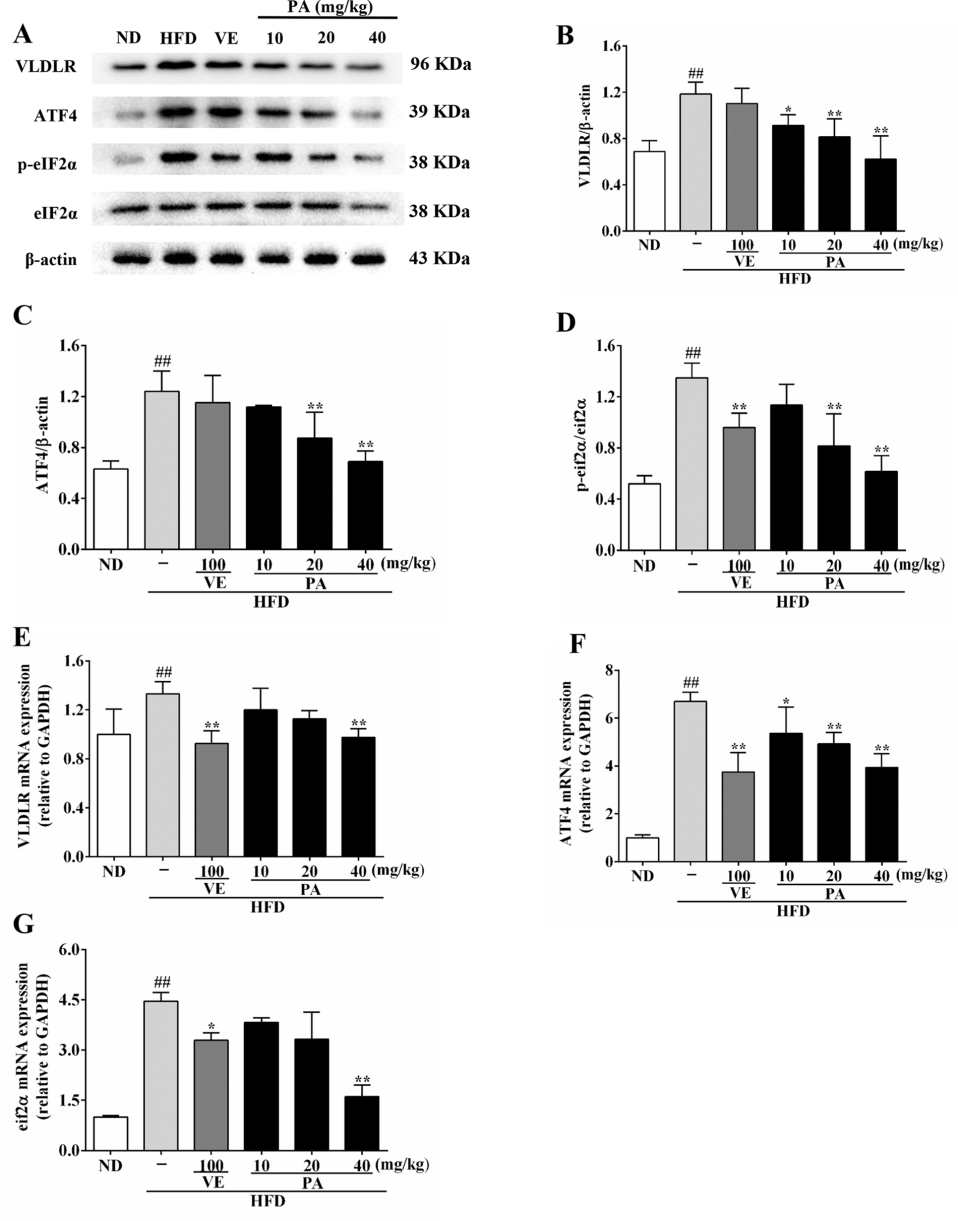
PA treatment attenuated HFD-induced VLDLR expression in rats. **(A)** Representative immunoreactive bands of eIF2α, p-eIF2α, ATF4, and VLDLR; **(B)** Ratios of VLDLR/β-actin, **(C)** ATF4/β-actin and **(D)** p-eIF2α/eIF2α; the relative expression levels of target proteins were normalized by β-actin; **(E)** mRNA expressions of VLDLR, **(F)** ATF4, and **(G)** eIF2α; the relative expression levels of target genes were normalized by GAPDH. Values were presented as mean ± SD (*n* = 3 per group). ^##^ *p* < 0.01 vs. ND group; *p < 0.05,**p < 0.01 vs. HFD group.

VLDLR plays a vital role in modulating VLDL-TG metabolism. To investigate the impact of PA on VLDLR expression, we measured the VLDLR and VLDLR-related indicators. Data in **Figures 5A–D** showed that the hepatic protein expressions of ATF4, VLDLR, and the ratio of p-eIF2α/eIF2α were promoted in HFD-fed rats. In contrast, VE and PA treatment dramatically demoted the expressions of these proteins, while there was no significant difference in VLDLR and ATF4 expression between HFD and VE treated groups. Data in **Figures 5E–G** showed that the hepatic mRNA expressions of ATF4, VLDLR, and eIF2α were significantly increased in HFD-fed rats, whereas these trends were completely inhibited by VE and PA treatment. These results suggested that PA may prevent hepatic steatosis by decreasing VLDLR expression and regulating VLDL metabolism.

### Effect of PA on ApoB100 Secretion in HFD-Fed Rats

**Figure 6:**
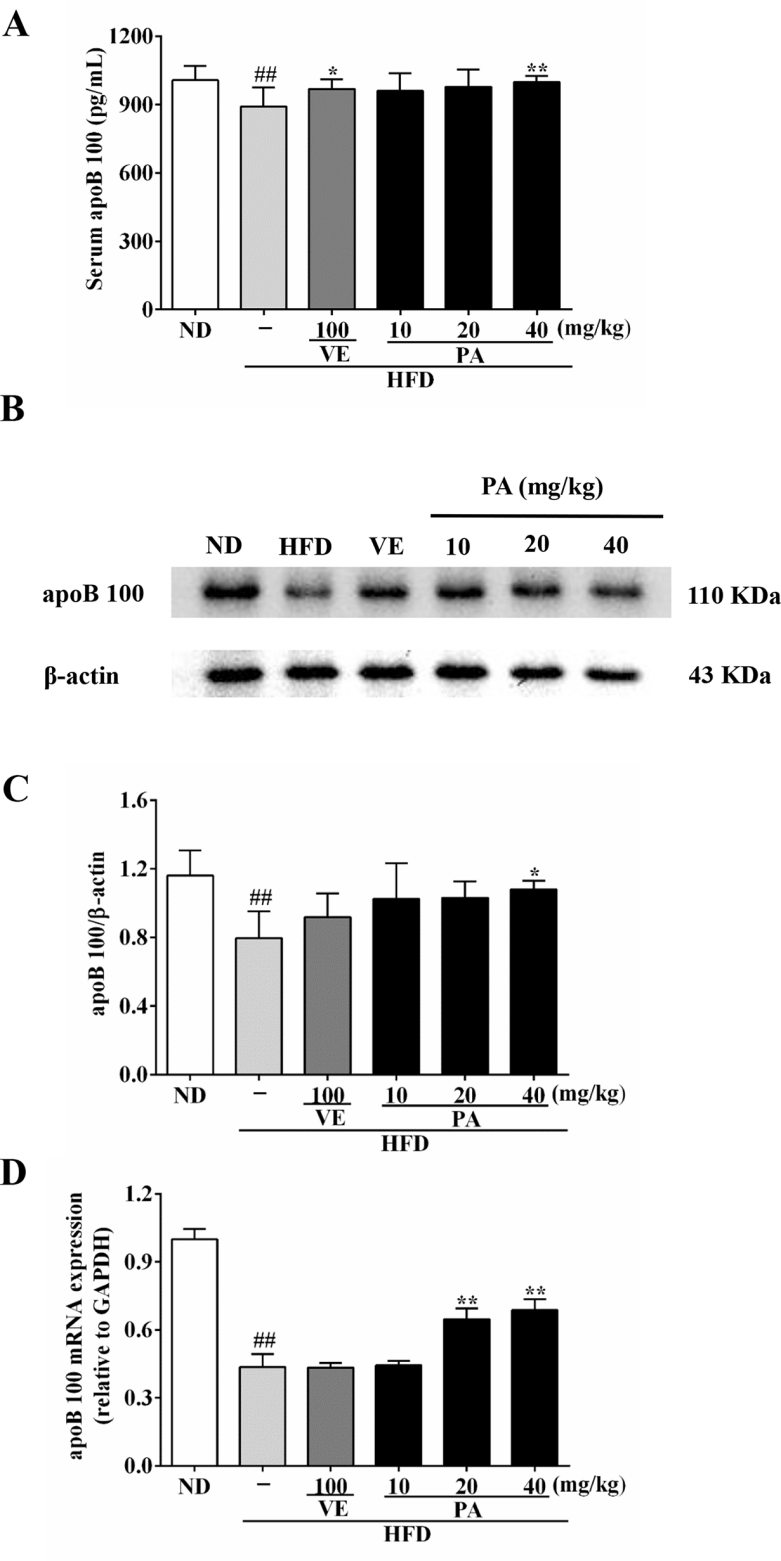
PA treatment attenuated HFD-induced apoB 100 reduction in rats. **(A)** Serum level of apoB 100 (*n* = 8 per group); **(B)** Representative immunoreactive band of apoB 100; **(C)** Ratio of apoB 100/β-actin; the relative expression level of target protein was normalized by β-actin (*n* = 3 per group); **(D)** mRNA expression of apoB 100; the relative expression level of target gene was normalized by GAPDH (*n* = 3 per group). Values were presented as mean ± SD. ^##^ *p* < 0.01 vs. ND group; *p < 0.05, **p < 0.01 vs. HFD group.

Serum and hepatic levels of apoB 100 were assessed to evaluate the effect of PA on apoB secretion. Data in **Figure 6** showed that the serum level as well as the hepatic mRNA and protein expressions of apoB 100 were markedly reduced after exposure to HFD. When rats were supplemented with VE and PA, the reduced serum level of apoB 100 as well as the protein and mRNA expressions in liver tissue of apoB 100 were enhanced. In addition, 40 mg/kg PA exhibited prominent effect comparable to other treated groups. No significant difference of the protein and mRNA expressions of apoB 100 was observed between the HFD and VE-treated groups. This suggested that under condition of PA treatment, serum and hepatic apoB 100 secretion was normalized and further beneficial for VLDL secretion.

### Effect of PA on MTP Level in HFD-Fed Rats

**Figure 7:**
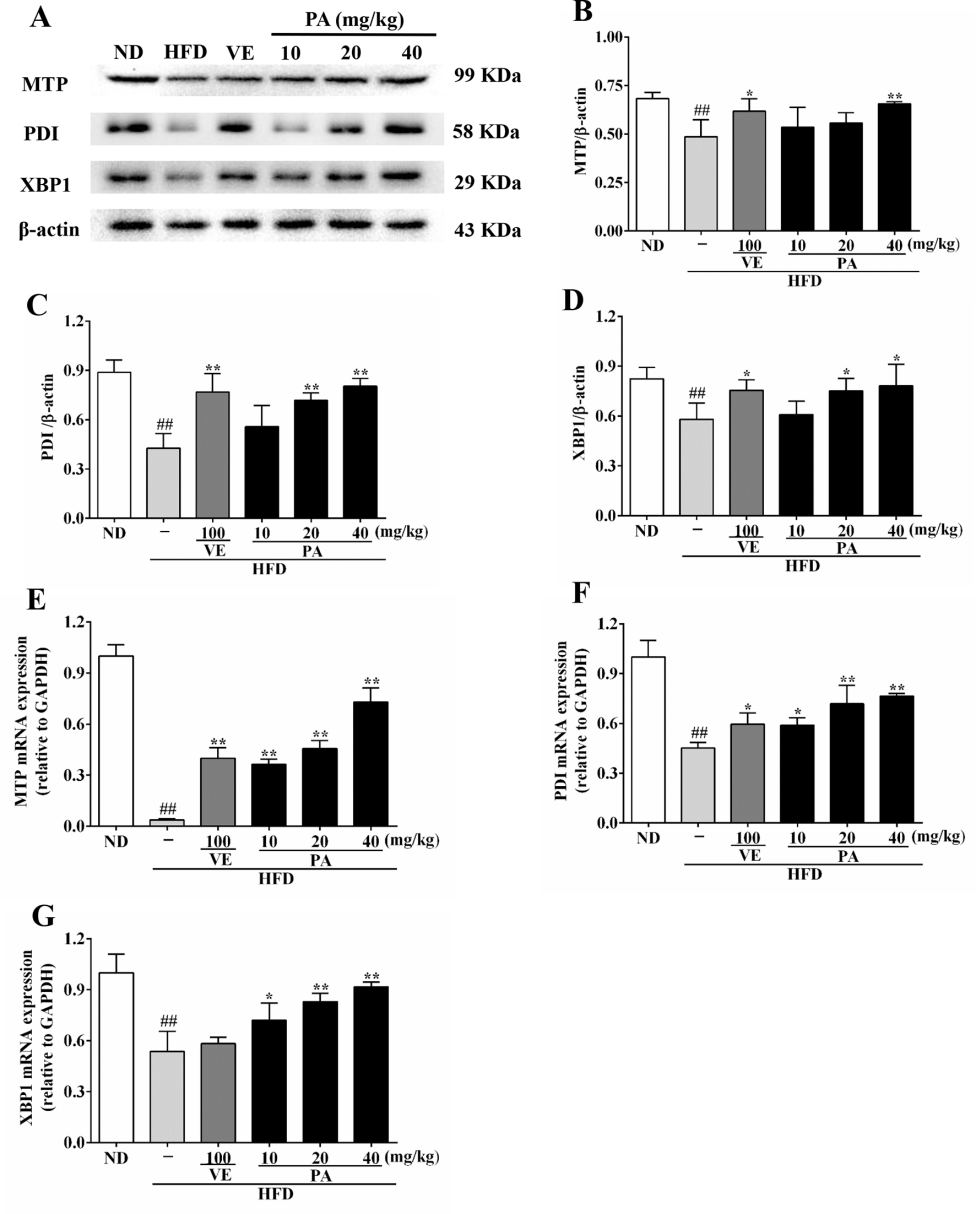
PA treatment attenuated HFD-induced MTP reduction in rats. **(A)** Representative immunoreactive bands of XBP1, PDI, and MTP; **(B)** Ratios of MTP/β-actin, **(C)** PDI/β-actin, and **(D)** XBP1/β-actin; the relative expression levels of target proteins were normalized by β-actin; **(E)** mRNA expressions of MTP, **(F)** PDI, and **(G)** XBP1; the relative expression levels of target genes were normalized by GAPDH. Values were presented as mean ± SD (*n* = 3 per group). ^##^ *p* < 0.01 vs. ND group; *p < 0.05, **p < 0.01 vs. HFD group.

To further understand the role of PA on MTP expression, we analyzed the MTP and related genes and proteins expressions in this study. Data in **Figure 7** showed that the chronic stimulation with HFD caused decreases of hepatic protein and mRNA expressions of XBP1, PDI, and MTP. However, these trends were dramatically attenuated by VE and PA administration. These results indicated that PA supplementation increased MTP level and restored VLDL secretion in HFD-fed rats.

## Discussion

The increasing intake of dietary lipid makes the occurrence of NAFLD widespread. *Pogostemon cablin*, a traditional healthy food and medicinal herb, was proved to possess hepatoprotective activity against lipid accumulation in rats. PA is a main effective component of *Pogostemon cablin*. However, its protective effect for the treatment of lipid deposition still remains elusive. In this study, it is firstly provided evidence that PA could alleviate HFD-induced hepatic steatosis by inhibiting lipid droplet formation and lipid accumulation in liver accompanied with reduced levels of TG, TC, FFA, AST, and ALT. Its key mechanism may be involved in suppressing ER stress and regulating VLDL metabolism.

ER is served as a main site of lipid synthesis and VLDL assembly. Previous studies have demonstrated hepatic ER stress in several animal models of steatosis, suggesting that ER stress may contribute to the induction of NAFLD (Ghemrawi et al., 2018). Elevation of FFA concentration and oxidative stress are common feature of NAFLD and proved to be tightly associated with ER stress. Excessive FFA not only activates cellular ER stress, but also causes oxidative stress by enhancing mitochondria-associated membranes (MAM) and increasing ROS production (Hager et al., 2012; Ghemrawi et al., 2018). Oxidative stress could perturb the redox status of ER lumen and inhibit protein folding, then acts as a trigger to ER stress (Ashraf and Sheikh, 2015). In response to ER stress, normal ER function in maintaining protein homeostasis becomes compromised, resulting in accumulation of unfolded or misfolded proteins and triggering unfolded protein response (UPR). During the response to ER stress, all three main brunches of UPR including PERK, IRE1, and ATF6 pathways are activated and mediates hepatic steatosis. GRP78 is an ER stress marker in liver. Under unstressed conditions, IRE1, PERK, and ATF6 are associated with GRP78 and remain inactive. Upon ER stress, GRP78 dissociates from these sensor proteins, and further actives PERK and IRE1, and regulates intramembrane proteolysis of ATF6 (As, 2018). In this study, after 4 weeks of PA administration, the increased levels of ROS and MDA were markedly decreased in HFD-fed rats. PA also increased the GSH, SOD, CAT, and VLDL levels in HFD-fed rats. Moreover, PA treatment decreased the protein and mRNA expressions of ER stress markers including GRP78, IRE1, PERK, and ATF6. This indicated that PA may restore VLDL secretion and attenuate hepatic steatosis by alleviating ER stress in HFD-induced NAFLD rats.

Recent data has revealed that the activated PERK–eIF2α–ATF4 pathway during ER stress induces hepatic steatosis *via* increase VLDLR by enhancing intracellular TG accumulation with VLDL uptake (Jo et al., 2013). VLDLR is a member of low-density lipoprotein receptor (LDLR) superfamily. It binds APOE-containing VLDL, which then converts into TG, leading to decrease lipid secretion and increase lipid accumulation (Kim et al., 2014). Previous study has demonstrated that inhibition of VLDLR upregulation can protect mice against hepatic steatosis induced by HFD feeding (Zaire et al., 2018). Upon dissociation from GRP78, PERK is activated by dimerization and autophosphorylation, triggering phosphorylation of the eIF2α. Furthermore, promotion of eIF2α halts global protein translation and selectively translates ATF4 mRNA (Lebeaupin et al., 2018). ATF4 is a well-known transcription factor that mediates PERK downstream pathway and functions to increase hepatic VLDLR expression (Jo et al., 2013). In our work, PA treatment significantly reduced the mRNA and protein expressions of eIF2α, ATF4, and VLDLR. These results demonstrated that PA is able to lower VLDL uptake by down-regulating VLDLR.

ER stress not only impacts the hepatocytes uptake of VLDL but also impairs VLDL secretion. VLDL synthesis is a two-stage process. The first step in VLDL assembly is the apoB synthesis within the ER lumen following by its lipidation by MTP and the inclusion of TGs into a lipid droplet. In the second step, bulk neutral lipid, especially TGs, are added to the VLDL precursors and form lipid-rich VLDL. ApoB100 is a major protein component of VLDL, accounting for approximately one third of total lipoproteins present in VLDL. Impaired apoB 100 synthesis results in reduced VLDL synthesis, which inhibits the transport of endogenous TG from the liver to the extrahepatic, leading to TG deposition in hepatocytes (Watts et al., 2017). Hepatic apoB100 synthesis and secretion is a complex process involving ER stress. Under conditions of ER stress, hepatic lipid synthesis and secretion are affected, making a significant proportion of newly synthesized apoB100 degraded *via* the ubiquitin-proteasome-dependent degradative pathway (Suzuki et al., 2012). Evidences showed that ER chaperone protein such as GRP78 increased accompanied by decreasing apoB100 secretion, suggesting that there is an inverse relationship between ER stress and apoB100 secretion (Ota et al., 2008). In addition, apoB100 secretion appears to be regulated by PERK and ATF6 pathways. Activated PERK pathway is found to impair apoB100 synthesis in glucosamine-treated cells (Qiu et al., 2009). Recent study has pointed out that ATF6α-knockout mice show enhanced hepatic steatosis caused by impaired formation of VLDL due to destabilized apoB100, whereas the exact mechanism of ATF6 in regulating apoB100 formation still remains unclear (Yamamoto et al., 2010). Following exposed to HFD, the serum level as well as the protein and mRNA expressions of apoB100 were decreased in rats. However, PA treatment altered apoB100 secretion in HFD-fed rats. This alteration of apoB100 secretion was proved to beneficial for VLDL assembly.

MTP is an ER-localized lipid transfer protein, plays a crucial role in lipoprotein assembly, and acts as a cofactor to apoB100 at both stages of VLDL synthesis. It is responsible for the lipidation of the nascent apoB protein and the transfer of neutral lipids between vesicles (Yao et al., 2013). PDI is a subunit of MTP necessary for normal MTP activity. Previous studies demonstrated that MTP activity is highly dependent on PDI expression and related to IRE1α- XBP1-PDI pathway (Wang et al., 2012). IRE1α-XBP1 induce PDI expression to increase MTP activity for VLDL assembly and secretion. IRE1 appears in mammal with two isoforms: IRE1α and IRE1β. IRE1α is a transmembrane protein that possesses endoribonuclease (RNase) activity, which is responsible for production spliced XBP1 (XBP1s). After activated by ER stress, IRE1α initiates unconventional splicing of XBP1 mRNA and translates it into a potent transcription factor (XBP1s) (Wang et al., 2018). In turn, XBP1s drives a large transcriptional program to adjust the ER’s protein-folding capacity according to the protein folding load in the ER lumen (Li et al., 2018). Our data showed that HFD-induced ER stress led to defective XBP1, MTP, and PDI expressions in rats. PA administration not only improved XBP1, PDI, and MTP expressions but also decreased IRE1α expression from ER stress. These results indicated that PA may be able to reduce steatosis by attenuating MTP down-regulation and restoring VLDL secretion.

In conclusion, this study provides compelling evidence to support that PA is effective in ameliorating hepatic steatosis caused by HFD through suppressing ER stress and regulating VLDL uptake, assembly, and secretion, which is associated with the regulation of VLDLR, apoB100, and MTP expression. Given the promising preclinical findings presented in this study, we suggest that PA might play a protective role as possible therapeutic agents acting on hepatic steatosis.

## Data Availability Statement

The datasets analyzed in this manuscript are not publicly available. Requests to access the datasets should be directed to liuyuhong@gzucm.edu.cn.

## Ethics Statement

The animal study was reviewed and approved by Animal experiment procedures were approved by the Ethics Committee for the Welfare of Experimental Animals of Guangzhou University of Chinese Medicine (No. 20181015002).

## Author Contributions

XW and YhL drafted and prepared the article. YhL and ZS conceived and designed the experiments; XW, NX, ML, QH, and JW performed experiments; YG, HL, and LC analyzed the data; YcL and XH prepared figures and tables.

## Funding

This work was supported by grants from Science and Technology Planning Project of Guangdong Province, China (2017A050506044), Guangdong Provincial Department of Education Feature Innovation Project (2016KTSCX018), Key Disciplines Construction Projects of High-level University of Guangdong Province, Key Program for Subject Research of Guangzhou University of Chinese Medicine (XK2018016 & XK2019002), and Characteristic Cultivation Program for Subject Research of Guangzhou University of Chinese Medicine (XKP2019007).

## Conflict of Interest

The authors declare that the research was conducted in the absence of any commercial or financial relationships that could be construed as a potential conflict of interest.

## Abbreviations

apoB 100, apolipoprotein B100; ALT, alanine aminotransferase; AST, aspartate transaminase; ATF4, activating transcription factor 4; ATF6, activating transcription factor 6; CAT, catalase; eIF2α, eukaryotic translation initiation factor 2α; ER, endoplasmic reticulum; FFA, free fatty acid; GRP78, glucose-regulated protein 78 kDa; GSH, glutathione; HFD, high fat diet; IRE1α, inositol-requiring transmembrane kinase/endoribonuclease 1α; MDA, malondialdehyde; MTP, microsomal triglyceride-transfer protein; NAFLD, nonalcoholic fatty liver disease; ND, normal diet; PA, patchouli alcohol; PDI, protein disulfide isomerase; p-eIF2α, phospho-eIF2α; PERK, protein kinase-like ER kinase; p-IRE1α, phospho-IRE1α; p-PERK, phosphor-PERK; ROS, reactive oxygen species; SOD, superoxide dismutase; TC, Total Cholesterol; TG, triglyceride; VE, vitamin E; VLDL, very low-density lipoprotein; VLDLR, very low-density lipoprotein receptor; XBP1, X box binding protein 1.
